# Very Small Embryonic-Like Stem Cells: Implications in Reproductive Biology

**DOI:** 10.1155/2013/682326

**Published:** 2013-02-13

**Authors:** Deepa Bhartiya, Sreepoorna Unni, Seema Parte, Sandhya Anand

**Affiliations:** Stem Cell Biology Department, National Institute for Research in Reproductive Health (ICMR), Mumbai, Maharashtra 400 012, India

## Abstract

The most primitive germ cells in adult mammalian testis are the spermatogonial stem cells (SSCs) whereas primordial follicles (PFs) are considered the fundamental functional unit in ovary. However, this central dogma has recently been modified with the identification of a novel population of very small embryonic-like stem cells (VSELs) in the adult mammalian gonads. These stem cells are more primitive to SSCs and are also implicated during postnatal ovarian neo-oogenesis and primordial follicle assembly. VSELs are pluripotent in nature and characterized by nuclear Oct-4A, cell surface SSEA-4, and other pluripotent markers like Nanog, Sox2, and TERT. VSELs are considered to be the descendants of epiblast stem cells and possibly the primordial germ cells that persist into adulthood and undergo asymmetric cell division to replenish the gonadal germ cells throughout life. Elucidation of their role during infertility, endometrial repair, superovulation, and pathogenesis of various reproductive diseases like PCOS, endometriosis, cancer, and so on needs to be addressed. Hence, a detailed review of current understanding of VSEL biology is pertinent, which will hopefully open up new avenues for research to better understand various reproductive processes and cancers. It will also be relevant for future regenerative medicine, translational research, and clinical applications in human reproduction.

## 1. Introduction

Stem cells have the capacity to self-renew as well as give rise to differentiated progeny. They have generated a lot of interest amongst the general public as well as the scientific fraternity because of their potential for regenerative medicine. Although this field of research has been associated with a lot of hype, it definitely holds a lot of hope when applied to reproductive health. Considerable research has gone into the differentiation of embryonic stem cells [1, 2] and even induced pluripotent stem cells [3] to generate synthetic gametes. The idea of generating gametes *in vitro* has tremendous applications in treatment of infertility and understanding gametogenesis and also as a source of gametes for therapeutic cloning and regenerative medicine. However, although male gametes generated from mouse embryonic stem cells *in vitro* resulted in the birth of pups, most of them suffered epigenetic defects [4]. Similar issues may surface when stem cells isolated from ovaries of reproductive age women [5] are used to generate oocytes. It appears to be a major shortcoming and one wonders if this research will find translation in the clinics. Other applications of stem cells in the field of reproductive health have also been reviewed including the treatment of reproductive diseases [6].

Recently few groups have succeeded in deriving pluripotent ES-like cultures using adult testicular biopsies of mice [7–9] and humans [10–13]. These pluripotent stem cells are autologous, embryo-free, patient-specific, and potentially safe for regenerative medicine with no associated sensitive ethical issues as compared to embryonic stem cells. Emerging literature suggests that it may be possible to derive similar ES-like cultures from ovarian tissues of mice [14], humans [15, 16], and other higher mammalian species including rabbits, monkeys, and sheep [17]. Zou et al. [18] successfully cultured female germline stem cells derived from both neonatal and adult ovary for several months *in vitro, *which when transplanted in busulfan treated mice led to the birth of normal pups. This demonstrated supremacy of the gonadal stem cells differentiated by the *in vivo* cues over *in vitro* manipulated ES cells to generate synthetic gametes. White et al. [5] recently showed that DDX4 expressing cells isolated from adult mouse and reproductive age women can be used to generate oocytes *in vitro* as well as *in vivo* after xenotransplantation in immunodeficient mice.

It was postulated that spermatogonial stem cells (SSCs) undergo dedifferentiation and result in ES-like colonies *in vitro* [13], but recent studies from our group demonstrated the presence of pluripotent, very small embryonic-like stem cells (VSELs) with high nucleocytoplasmic ratio and nuclear Oct-4 in adult human testis [19] and ovary for the first time [17]. We propose that rather than dedifferentiation of SSCs as earlier postulated, it may be possible that the VSELs *per se* expand to give rise to ES-like colonies *in vitro *[20]. Their presence in few numbers in adult gonadal tissue biopsies may explain the poor success of ES-like colonies derivation *in vitro *from gonadal tissue biopsy. 

VSELs are the primordial germ cells that migrate into the gonadal ridge during early embryonic development and persist into adulthood, as also suggested by deFelici [21]. However, there is a disparity in the size of migrating PGCs (15–20 um) and VSELs (1–3 um); thus, more studies are needed to better understand whether the VSELs are similar or more primitive to PGCs. According to the existing school of thought, PGCs may give rise to pluripotent stem cells *in vitro* but they do not behave as stem cells *in vivo*, and later on during fetal development the true stem cell population of SSCs appears in the testis that divides throughout life giving rise to waves of spermatogenesis [22]. Similarly, Byskov et al. 2011 [23] have also suggested that ovary may have cells with stem-like characteristics which may be provoked to enter differentiation pathway into oocytes, at least *in vitro*. As evident a lot of misperception exists on our basic understanding of gonadal stem cells. 

An introduction to gonadal stem cells, namely, VSELs and their possible role during premeiotic expansion of germ cells during gametogenesis and their relevance to reproductive and cancer biology are the focus of the present paper. In conclusion, we will summarize the possible translational applications of this emerging and exciting field of research. 

## 2. Very Small Embryonic-Like Stem Cells (VSELs) 

Pluripotent VSELs (Oct4^+^, SSEA1^+^, Sca1^+^, Lin^−^, CD45^−^) were first reported by Ratajczak and group in adult mice tissues [24, 25], the highest numbers being in brain, kidneys, muscles, pancreas, and bone marrow [26]. These are diploid cells with high telomerase activity, express other pluripotent (Rex-1, Nanog, SSEA, and Klf-4) and germ cell (Mvh, Stella, Fragilis, Nobox and Hdac-6) markers, and decrease in numbers with age [27]. Like embryonic stem cells, they do not express MHC class I and HLA-DR antigens and are also negative for mesenchymal stem cell markers like CD90^−^, CD105^−^, and CD29^−^. They are very small in size (3–5 um) and have a large nucleocytoplasmic ratio, large nuclei with abundant euchromatin, and an open chromatin structure for Oct-4 and Nanog promoter [28]. Oct-4 expression at mRNA and protein level in VSELs has been confirmed using sequence specific primers. VSELs have the ability to differentiate into three germ layers *in vitro*; however, unlike ES cells, VSELs neither complement during blastocyst development nor form teratomas in immunodeficient mice [29]. Attempts have been made to propagate them on feeder layers, but they do not self-renew as easily as the established embryonic stem cell lines possibly do because of altered methylation status of some developmentally crucial genes. Similarly VSELs have also been isolated from human umbilical cord blood, mobilized peripheral blood, and adult bone marrow by flow cytometry as CD133^+^, lin^−^, CD45^−^ [30] and also by the differential centrifugation method [31, 32]. 

VSELs are descendants of epiblast stage pluripotent stem cells. They get deposited in various body organs including the gonads in early stages of development, as a quiescent stem cell population which possibly serves as a back up to the tissue committed stem cells (TCSCs). These two populations of stem cells (VSELs and TCSCs) together are responsible in bringing about tissue renewal, homeostasis, and regeneration after injury throughout life and decrease in number with age. The coexistence of two stem cell populations (the more primitive being quiescent and the progenitor being more rapidly dividing) has been recently proposed by Li and Clevers [33]. VSELs are the DNA label-retaining (e.g., BrdU), quiescent stem cells with a lower metabolic state whereas the tissue committed stem cells divide actively and do not retain DNA label over time. They are highly mobile, respond to the SDF-1 gradient, and enter into circulation in case of any injury to bring about regeneration and homeostasis. They are also considered as a missing link to support the germline hypothesis of cancer development [34, 35]. The clinical potential of VSELs, isolated from cord blood or bone marrow by flow cytometry, is just beginning to emerge. In various disease models like myocardial infarct [36, 37], stroke [38], skin burn injury [39], neural regeneration [40], and so forth, these cells get mobilized into circulation within 24 hours. For myocardial regeneration, the VSELs are very efficient to improve LV ejection fraction and attenuation of myocardial hypertrophy [37]. As they become scarce with age, regeneration becomes inefficient resulting in age-related disease manifestations.

## 3. Localization of VSELs in Mammalian Gonads

Our group has demonstrated the presence of VSELs for the first time in their natural somatic niche *in situ* in adult testicular and ovarian tissue collected from prostate cancer patients and perimenopausal women, respectively. These VSELs were localized in the basal layer of cells adjacent to the basement membrane in seminiferous tubules [19] and were found interspersed with the ovarian surface epithelial cells [17]. Similarly VSELs have also been observed in adult mice gonads [20], whereas the ovarian VSELs have been detected in scraped ovarian surface epithelium in rabbits, sheep, and monkey [17] and also in mouse ovary [41] by our group. Thus, the presence of VSELs in gonadal tissue appears to be evolutionarily conserved.

### 3.1. Oct-4 as a Pluripotent Marker to Study VSELs

Oct-4, also designated as Oct-3 or POU5F1, is present as a maternal transcript in mature oocytes and besides being the gatekeeper in the beginnings of mammalian development [42] and pluripotency of inner cell mass in blastocysts, it is also a cell fate instructor through gene dosage effect [43] and is essential for primordial germ cell survival [44]. Oct-3/4 expression has also been associated with germ cell tumors and gonadoblastoma. Oct-4 gene is located on chromosome 6 and has five exons. It encodes two main variants by alternative splicing, namely, Oct-4A and Oct-4B which differ from each other in that exon 1 is present only in Oct-4A. The two transcripts give rise to 360 aa and 265 aa, respectively, of which 225 aa of C-terminal are identical. In contrast to Oct-4A, Oct-4B is not responsible for pluripotency [45]. 

Published literature on Oct-4 in somatic stem cells has confused stem cell researchers [46–48] because of the presence of several pseudogenes and alternatively spliced transcripts [46, 49]. Thus, a careful designing of primers for RT-PCR analysis and proper selection of antibodies becomes essential to detect specific transcripts. Also, a careful selection of Oct-4 antibodies is essential to detect pluripotent stem cells [47]. We used a polyclonal Oct-4 antibody that enabled the simultaneous identification of VSELs with nuclear Oct-4 and tissue committed stem cells, namely, SSCs and OGSCs with cytoplasmic Oct-4. In addition, careful selection of primers for Oct-4A and total Oct-4 for Q-PCR studies has helped us generate interesting results [17, 19, 20, 41].

Presence of Oct-4 positive VSELs in adult gonads and other body tissues contradicts the earlier views proposed by Jaenisch's group [50, 51] that an active pluripotency Oct-4 network exists only in embryonic and induced pluripotent stem cells. No abnormalities in homeostasis or regeneration were observed by them even after silencing Oct-4 gene in various tissues like intestine, bone marrow, hair follicle, liver, CNS, and so forth in 8-week-old mice. On the basis of the results, they proposed that Oct-4 is dispensable for functions of somatic cells. Berg and Goodell [52] authored a commentary on their work and had speculated that it may be possible for stem cells that were not directly tested in the experiments to have brought about the regeneration. In agreement with their view, it is felt that the regeneration may have occurred by the VSELs which get mobilized from the bone marrow into the circulation, in response to the injury. Thus, although the tissue specific Oct-4 was deleted, normal regeneration and homeostasis were observed in the young 8–10-week-old mice. It would be interesting to carry out similar studies in old mice (>12–14 months) having probably reduced number of VSELs and to observe whether regeneration occurs or not. 

Indeed presence of VSELs have confused biologists in several other instances as well; for example, Tilly's group [53] and Nayernia et al. [4] concluded that bone marrow could be a possible source of female and male germ cells, respectively—leading to a flurry of scientific debate in the literature. Similarly, cells with early cardiac markers have been reported to be present in the bone marrow [54]. All these results are easily explained on basis of VSELs which are pluripotent stem cells and can differentiate into any kind of differentiated progeny depending on the body's need. 

### 3.2. VSELs in Adult Testicular Tissue

We have documented that an adult testis harbors a novel population of pluripotent VSELs (with nuclear Oct-4A) which are more primitive to A_dark_ SSCs (with cytoplasmic Oct-4B). The VSELs possibly give rise to A_dark_ SSCs which in turn undergo clonal expansion as evident by the presence of cytoplasmic bridges between the rapidly dividing cells [19]. Oct-4 is not immuno-localized in more differentiated male germ cells.

**Figure 1:**
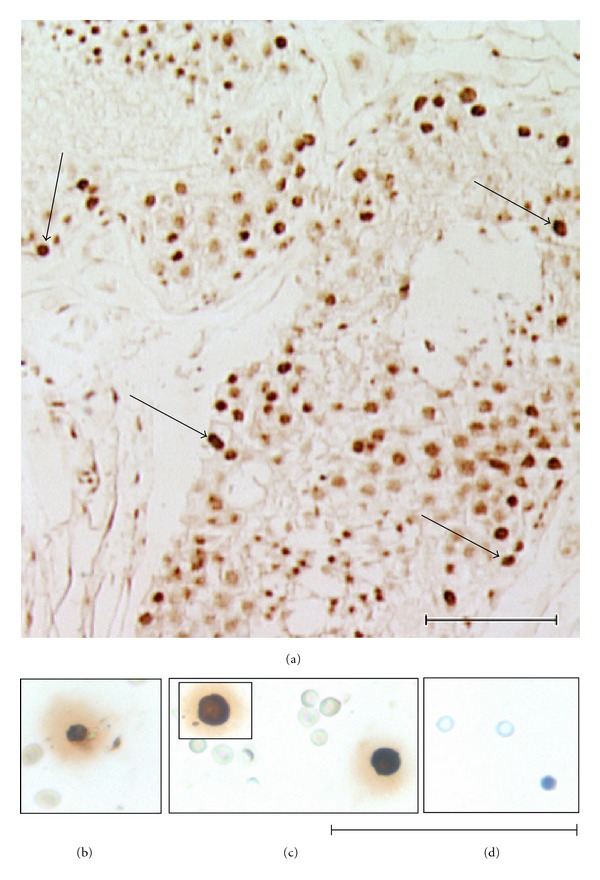
Immunolocalization of 5-methyl cytosine on adult human testicular section and peri-menopausal ovary surface epithelium smear (using standard protocol published earlier, [55]). Note dense staining in the spermatogonial stem cells (SSCs, arrow, (a)) while the spermatocytes showed minimal staining. In few tubules spermatids showed positive staining. Similarly the ovarian germ stem cells (OGSCs) stain positive ((b) and (c)) Negative control (d). The results indicate that A_dark_ SSCs in testis and OGSCs in ovaries, derived by asymmetric cell division of VSELs undergo nuclear reprogramming associated with extensive methylation—suggesting that similar basic stem cell biology exists in both the sexes. Scale bar = 20 *μ*m.

The characteristic dark stained nuclei in A_dark_ SSCs is easily explained on the basis of stem cell biology. VSELs have abundant open euchromatin and the differentiated cells that arise by asymmetric cell division undergo extensive reprogramming and compaction of chromatin (by DNA methylation) which may result in a dark nuclear appearance, a characteristic of the A_dark_ SSCs [19]. Chromatin compaction occurs by DNA methylation wherein cytosine gets methylated and enables DNA to maintain similar sequence but genes get silenced or activated. This process can be studied using a simple immunolocalization procedure. More direct evidence and multicolor colocalization studies need to be carried out to prove this hypothesis but the preliminary immunolocalization study carried out using monoclonal 5-methyl cytosine antibody (source: Calbiochem, Merck, Millipore) has yielded interesting results (Figure 1(a)). Staining was predominantly observed in A_dark_ SSCs indicating on extensive nuclear reprogramming in the progenitor stem cells which arise by asymmetric cell division from the pluripotent VSELs (with abundant euchromatin).

### 3.3. VSELs in Adult Ovarian Tissue

Careful scraping of ovarian surface epithelium in rabbits, sheep, monkey, and perimenopausal women resulted in the detection of VSELs (1–3 um) and also ovarian germ stem cells (OGSCs; 5–7 um). The VSELs were smaller than RBCs, had high nucleocytoplasmic ratio, abundant euchromatin, nuclear OCT-4, cell surface SSEA-4 and other pluripotent markers [17]. Interestingly, H & E staining of the stem cells in scraped OSE resulted in the visualization of OGSCs with dark stained nuclei [17], possibly signifying similar stem cells biology like A_dark_ SSCs and exhibited nuclear staining for 5-methyl cytosine (Figures  1(b)–1(d)). Three-week culture of these stem cells gave rise to putative oocyte-like structures, embryo-like structures, neuron-like structures, ES-like colonies, and embryoid-like bodies, signifying the pluripotent to totipotent nature of the stem cells [17]. 

## 4. Role of VSELs during Gametogenesis

Gametogenesis, a process by which haploid gametes are produced from diploid germ cells in the gonads, ensures transmission of genetic information from generation to generation and thus the continuation of species. The primordial germ cells (PGCs) of epiblast stage embryo colonize into the gonadal ridge in the undifferentiated gonad and differentiate into female or male germ cell precursors. These PGCs possibly persist as VSELs in adult gonads and undergo asymmetric cell divisions throughout life to self-renew and give rise to tissue committed gonadal stem cells, namely, A_dark_ SSCs in the testis and OGSCs in the ovary. 

### 4.1. Spermatogenesis

Undifferentiated SSCs maintain a stable diploid population of germ cells and produce differentiating spermatogonia, which finally enter meiosis and give rise to spermatocytes which differentiate and produce sperm throughout life. A comprehensive review on various aspects of self-renewal, proliferation, and differentiation of SSCs was recently published [56, 57] highlighting the dearth of our present knowledge. 

**Figure 2:**
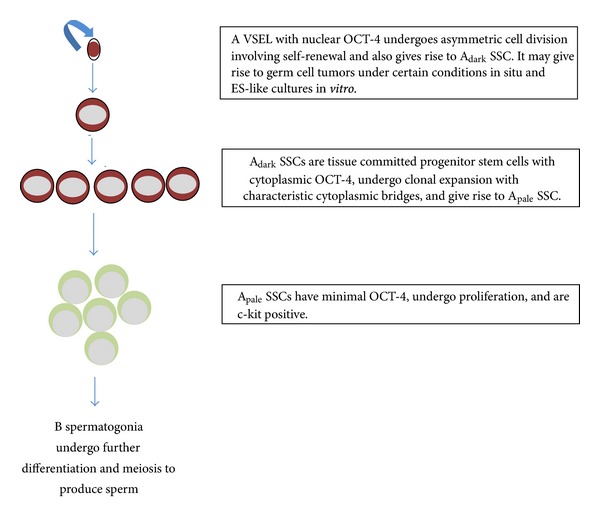
VSELs are implicated during human spermatogenesis. The relationship between VSELs and SSCs during premeiotic expansion of germ cells is depicted.

Difference of opinion exists in our understanding of kinetics of proliferation of SSCs in the humans based on the earlier reports of Clermont [58] and the recently proposed scheme by Ehmcke and Schlatt [56]. Clermont suggested that the A_dark_ SSCs undergo regular mitotic divisions whereas A_pale_   spermatogonia divide only once whereas Ehmcke and Schlatt propose that spermatogenesis starts with the division of a pair of A_pale_ spermatogonia and A_dark_ SSCs divide very rarely. They further suggest that A_dark_, A_pale_ and B spermatogonia do not form mixed pairs or chains and none of the premeiotic germ cell types undergo unequal divisions. Detection of VSELs in the adult testis adds another dimension to this current school of thought since they are implicated during pre-meiotic expansion of testicular germ cells. Various events like asymmetric cell division, self-renewal, clonal expansion, and proliferation as they occur during spermatogenesis have been further clarified (Figure 2). Till recently, the description of SSCs in primates has been based on studies conducted on histological sections, whole mount preparations, and so forth. An urgent need is felt to study the expression and localization of various growth factors and cytokines in the testicular compartment with respect to various stages of proliferation and differentiation. This kind of stage-specific analysis of germ cell markers approach will help define and dissect out mitotic, meiotic, and postmeiotic germ cell processes leading to improved translational opportunities as suggested earlier also [59].

### 4.2. Oogenesis

Even after decades of research, reproductive biologists are still confused whether the ovary has a fixed number of follicles at birth which diminish with age and menopause is associated with a dramatic decline in number of follicles or there is a continuous renewal of follicles throughout adulthood just like sperm in testis (recently reviewed in favor of postnatal oogenesis by Woods and Tilly [60] and in favor of a fixed number of eggs by Notarianni [61]). 

VSELs have been reported in the scraped surface epithelium of rabbit, sheep, monkey, and perimenopausal human ovary [17]. It is interesting to mention here the work published by Szotek and group [62] which showed the presence of a population of stem cells in mice OSE that retains label for more than four months—indicating quiescence with asymmetric label retention. In addition to the VSELs in the scraped OSE, slightly bigger cells with more cytoplasm that express cytoplasmic Oct-4 and minimal SSEA-4 also exist—which are the progenitor ovarian germ stem cells (OGSC), comparable to A_dark_ SSCs in the testis. Later the OGSCs get surrounded by pregranulosa cells that develop by epithelial mesenchymal transition of epithelial cells, resulting in PF assembly as suggested recently by us [17]. 

A recent report by Byskov and group [23] found no evidence for the presence of oogonia in the adult human ovary after their initial clearance in first two years of postnatal life. However, the archived tissues that were used to arrive at this conclusion were fixed in formaldehyde and 30–40 um sections were used for immunostaining. Such an approach will never detect the VSELs (being 3–5 um in size) and could have resulted in negative results [63]. The choice of fixative and its effect on immunolocalization results has been discussed earlier [19, 64]. In contrast to their results, confocal microscopy studies on scraped surface epithelium from rabbit, monkey, sheep, and human ovaries [17] demonstrated the presence of a distinct population of stem cells with Oct-4 (both nuclear and cytoplasmic) and cell surface SSEA-4. Similarly, Zhang et al. [65] generated experimental evidence that no mitotically active female germline progenitors exist in adult mouse ovaries. However, here the choice of cell surface marker DDX1 to isolate the progenitors is an issue [66, 67]. Basically, using DDX-1 as a marker they used 10–15 *μ*m cells for their study, whereas VSELs range between 3–5 *μ*m in size. Thus, rather than using DDX-1, SSEA-1 in mouse (SSEA-4 in humans) may be a better cell surface marker to isolate pluripotent ovarian stem cells. 

Intriguingly Byskov et al. [23] in their paper discussed the results of Liu et al. [68] who also found no evidence for the presence of oogonia in normal adult human ovaries, neither early meiosis-specific or oogenesis-specific mRNAs nor immunohistochemical markers for oogonia or meiosis. But a closer scrutiny of the published results of RT-PCR and immunolocalization studies [68] shows Oct-3/4, DMC1, and SCP3 in adult ovary (although much less as compared to fetal samples)—which cannot be ignored. RT-PCR data also fail to discriminate between cells having a low level of expression compared to a scenario where few cells exist with high expression. In the light of these concerns, the existence of stem cells and oogonia in adult human ovary needs to be re-evaluated. These discrepancies and ambiguous biological conclusions based on technological limitations need to be resolved and the concept of presence of stem cells and postnatal oogenesis in adult ovary should be understood soon. 

## 5. Effect of Aging on VSELs in Mammalian Gonads

The stem cells exist in a specialized microenvironment provided by the somatic cells termed as the “niche”. This term was first coined by Schofield in 1978 for the mammalian hematopoietic system [69] and now is discussed in context to various tissues. The niche ensures normal functioning of stem cells and regulates specific properties like self-renewal, pluripotency, quiescence, and ability to differentiate. It gets compromised with age and results in reduced homeostasis and regeneration ability of stem cells [70]. 

### 5.1. Effect of Aged Niche in Males

An age related reduction in both quality and quantity of sperms in mice and men is possibly because of compromised niche rather than the reduction of stem cell potential [71, 72]. When SSCs from young, fertile male mice are transplanted into 1-and 2-year-old atrophied testis, only 1-year-old testis showed regeneration, thus indicating age-related alterations in somatic cells can impair spermatogenesis. The impaired ability of Sertoli cells to respond to FSH and reduced production of GDNF with age [73] explains the age-related decline in fertility. 

### 5.2. Effect of Aged Niche in Females

 Menopause is the age-related cessation of ovarian function indicating the end of fertile phase of a woman's life. The mammalian ovary is believed to be endowed with a fixed number of eggs and a sudden loss of PF results in menopause in women [74, 75] and infertility in mice [76]. However, the proponents of postnatal oogenesis in females propose that menopause may actually be the result of compromised somatic niche (comprised of ovarian epithelial cells), which does not allow stem cells to undergo self-renewal, differentiation, and follicular assembly to form PF *in situ* [77, 78]. OSE stem cells from anovulatory postmenopausal ovaries have the capacity to differentiate *in vitro* into oocytes [79] and the immune system could be responsible for termination of follicular renewal *in vivo* [80]. Estradiol secretion by the ovarian tissue is reduced *in vitro* after being exposed to oncotherapy and also if collected from aged ovaries [77] indirectly indicating a compromised somatic environment which may restrict stem cells to undergo follicular assembly and thus result in menopause. Lee et al. [81] reported that BMT can restore long term fertility in preclinical mouse model of chemotherapy induced premature ovarian failure. Niikura [82] have shown that ovarian stem cells from aged mice ovary into a young host result in the resumption of oogenesis. The three-week culture studies of stem cells collected by scraping OSE of menopausal ovary suggest that once the *in vivo* inhibitory cues are withdrawn, the stem cells differentiate into oocytes-like structures, embryos, ES-like colonies, and EB-like structures *in vitro*, demonstrating their pluripotent nature. This has been a consistent observation not only in case of humans but also in other mammals like rabbit, sheep, and monkey [17].

## 6. VSELs and Cancers

VSELs are the possible precursors to cancer stem cells [34, 35]. It is a well-known fact that incidence of malignant tumors increases with old age [83], in both animals and humans. A change in the aged somatic niche disrupts the stem cell biology and VSELs possibly undergo a symmetric cell division resulting in tumor rather than their quiescent nature and asymmetric cell divisions under normal conditions in a younger niche as has been suggested earlier also [84]. Oct-4 is a well-established marker for diagnosis of carcinoma *in situ *(CIS), neoplastic gonadoblastoma, and invasive germ cell tumors in adults [85, 86]. Cools et al. [87, 88] concluded that gonadoblastomas, gonadal maturation delay, and early germ cell neoplasia in patients with under-virilization syndromes, have Oct 3/4 positive germ cells in dysgenetic gonads. 

The connection of stem cells (VSELs) with ovarian cancers is based on the published literature and circumstantial evidence but is not yet well accepted by the scientific community. 90% of ovarian cancers (most lethal amongst the gynecological malignancies) arise from OSE (which also houses the VSELs)! Incessant ovulation hypothesis suggests that continuous ovulation (without associated apoptosis) subjects OSE to transformation events and damaged cells are retained leading to cancer [89]. Overexpression of FSHR is also observed in OSE in cancer tissues as compared to normal OSE—that may activate oncogenic pathways leading to cancer [90]. Chen et al. [91] suggested that neither incessant ovulation nor FSHR present in OSE is required for inducing ovarian tumors. They argued that FORKO mice have high circulatory levels of FSH and LH, ovarian androgens are elevated, estrogens are very low, no FSHR and still have high incidence (>90%) of ovarian tumors by 12 months of age. These mice have endocrine profile similar to postmenopausal women, are infertile, never ovulate, and have no FSHR but still develop cancers. Thus, the only consistent observation is that ovarian cancers are more frequent in menopausal women, where possibly the VSELs residing in a compromised somatic niche may be implicated in tumor growth. It will be interesting to study VSELs in FORKO mice and especially age related changes in the somatic niche that triggers uncontrolled proliferation of these stem cells that lead to cancer. 

## 7. VSELs as Autologous Source of Pluripotent Stem Cells for Regenerative Medicine

Derivation of ES-like cultures using adult gonadal tissue from mice and humans has recently been reported. This has resulted in a lot of excitement since adult gonads may be a novel “autologous”, non-embryonic source of pluripotent stem cells in contrast to human embryonic stem cells where issues regarding immune rejection exist during cell-based therapies in future. Moreover, they may also be superior to induced pluripotent stem cells (iPS) since they are derived from a very quiescent stem cell population and are thus “young” cells with long telomeres that could be isolated from an aged body, in contrast to iPS cells which are derived from terminally differentiated somatic skin fibroblasts (with shortened telomeres) that tend to accumulate DNA mutations over time. This is in accordance with the “disposable soma” theory proposed by Kirkwood et al. [92], which suggests that investments into maintenance are higher in germline cells and get down regulated as “too costly” in somatic cells. 

However, extensive research needs to be undertaken to establish technology to obtain *in vitro* expansion of VSELs similar to embryonic stem cells. Attempts in the field have resulted in successful ES-like culture from testicular tissue in mice [7–9] and men [10–13]. These cultures have been characterized using various pluripotent markers and exhibit some differences compared to embryonic stem cells. They have the ability to differentiate into three germ layers but result in small teratoma formation [10, 11] after injecting more number of cultured ES-like cells into immuno-compromised mice. This may actually be good reason to prefer these cells over embryonic stem cells for regenerative medicine [13] and could be because of the epigenetic differences between VSELs (from which ES-like cultures were derived during testicular cultures) and embryonic stem cells especially related to insulin growth factor [93].

## 8. Future Perspectives

Application of VSELs to improve reproductive health needs to be established. We have recently studied the differential effect of busulfan on the relatively quiescent VSELs versus rapidly dividing germ cells in adult mice gonads (unpublished results). The VSELs were found to be resistant to the treatment and this opens up newer and exciting avenues for fertility preservation. The VSELs are localized in OSE and several investigators have reported extensive proliferation in OSE in response to PMSG [94]; we need to understand these published results in context of stem cells. Also a better understanding of VSELs will help manage menopause, infertility, and reproductive diseases. How these stem cells are implicated in PCOS and POF patients and so forth is altogether a new field of research with direct bearing on a woman's health that requires further investigation.
